# Mitochondrial permeability transition pore as a selective target for anti-cancer therapy

**DOI:** 10.3389/fonc.2013.00041

**Published:** 2013-03-08

**Authors:** Dong H. Suh, Mi-Kyung Kim, Hee S. Kim, Hyun H. Chung, Yong S. Song

**Affiliations:** ^1^Department of Obstetrics and Gynecology, Seoul National University College of MedicineSeoul, South Korea; ^2^Cancer Research Institute, Seoul National University College of MedicineSeoul, South Korea; ^3^Major in Biomodulation, World Class University, Seoul National UniversitySeoul, South Korea

**Keywords:** apoptosis, mitochondria, mitochondrial permeability transition pore, cancer, anti-cancer therapy

## Abstract

Mitochondrial outer membrane permeabilization (MOMP) is the ultimate step in dozens of lethal apoptotic signal transduction pathways which converge on mitochondria. One of the representative systems proposed to be responsible for the MOMP is the mitochondrial permeability transition pore (MPTP). Although the molecular composition of the MPTP is not clearly understood, the MPTP attracts much interest as a promising target for resolving two conundrums regarding cancer treatment: tumor selectivity and resistance to treatment. The regulation of the MPTP is closely related to metabolic reprogramming in cancer cells including mitochondrial alterations. Restoration of deregulated apoptotic machinery in cancer cells by tumor-specific modulation of the MPTP could therefore be a promising anti-cancer strategy. Currently, a number of MPTP-targeting agents are under pre-clinical and clinical studies. Here, we reviewed the structure and regulation of the MPTP as well as the current status of the development of promising MPTP-targeting drugs.

## Introduction

Mitochondria are intracellular organelles essential not only for energy production but also for various types of cell death including apoptosis, necrosis, and mitotic catastrophy via mitochondrial outer membrane permeabilization (MOMP) (Martel et al., 2012). Well-established central roles of mitochondria in intrinsic apoptotic pathway are involved in cytotoxic effect of many of chemotherapeutic agents (Wenner, 2012). The mitochondria-mediated intrinsic pathway is initiated by various stimuli, such as high levels of cytoplasmic Ca^2+^, reactive oxygen species (ROS), the activation of pro-apoptotic Bcl-2 family proteins, or UV damage (Dejean et al., 2010; Shoshan-Barmatz and Ben-Hail, 2012). In response to the various stimuli, mitochondria release apoptogenic proteins like cytochrome c and Smac/Diablo, which are consistently shown to be important for the propagation of following apoptotic cascades to the final destruction of the cell (Youle and Strasser, 2008). It is generally believed that bioenergetic and biosynthetic changes in cancer cells contributing to the typical tumor characteristics of resistance are associated with the alteration of mitochondria in cancer cells (Gogvadze et al., 2009a). Accordingly, mitochondria have emerged as effective targets for novel anti-cancer agents, so called, mitocans (Neuzil et al., 2006).

Release of apoptogenic protein, particularly cytochrome c, is considered “a point of no return,” resulting in apoptotic cell death (Gogvadze et al., 2009a). This commitment step of apoptosis, release of cytochrome c from the intermembrane space, seems to largely depend on MOMP. Moreover, as apoptotic machinery of cancer cell mitochondria is structurally and functionally different from that of their normal counterparts (Fulda et al., 2010), mitochondrial-targeted agents are expected to have a tumor selectivity. Although the mechanisms underlying MOMP remain to be determined, it appears to be clear that various kinds of protein complexes on mitochondrial membranes, including Bcl-2 family proteins, orchestrate the final apoptotic responses such as DNA fragmentation and blebbing of the plasma membrane (Dejean et al., 2010). Among them, the mitochondrial permeability transition pore (MPTP) is thought as the most viable model of mitochondrial channels which account for the MOMP (Mathupala et al., 2006).

Here, we provide a brief review of the structure and regulation of the MPTP, followed by comprehensive review of the developmental status of promising MPTP-targeting drugs.

## The concept of the mitochondrial permeability transition pore

Mitochondrial permeability transition (MPT) is the sudden permeabilization of the inner mitochondrial membrane (IMM) in response to a noxious stimulus such as oxidative stress, Ca^2+^ overload, hypoxia, and cytotoxic drugs (McCommis and Baines, 2012). MPT is thought to occur after the opening of the MPT pore (MPTP), although the composition of MPTP is not clearly understood yet (Nakagawa et al., 2005). The opening of MPTP causes depolarization of the IMM and swelling of the matrix space, which results in nonspecific rupture of the outer mitochondrial membrane (OMM) due to larger surface area of the IMM than the OMM (Kinnally and Antonsson, 2007). Despite much debate on the role of MPTP in the mitochondrial pathway of apoptosis, there are several lines of evidence for the structure and regulation of MPTP in apoptosis.

## Structure of the MPTP

Despite accumulating evidence for the molecular constituents of the MPTP, the proteins responsible for pore formation have not been fully identified. Traditionally, MPTP has been regarded as a multimeric complex, which putatively consists of the voltage dependent anion channel (VDAC) in the OMM, the adenine nucleotide translocase (ANT) in the IMM, cyclophilin D (CyP-D)—a mitochondrial matrix protein that exhibits peptidylprolyl cis-trans-isomerase activity, and some other proteins such as hexokinase (HK) (Mathupala et al., 2006; Gogvadze et al., 2009a). However, several studies reported that none of them might be part of the pore, even if ANT and CyP-D were MPTP regulators (Kokoszka et al., 2004; Baines et al., 2005, 2007).

### VDAC as a part of the MPTP

**Figure 1:**
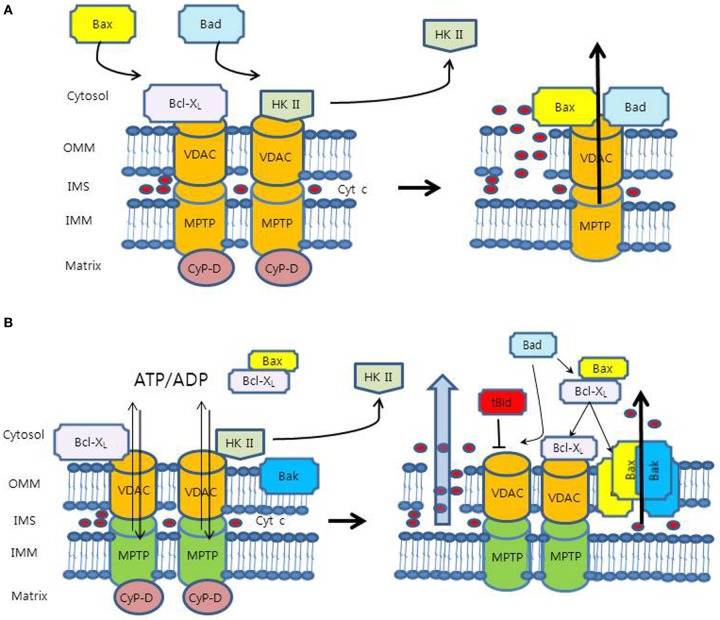
**Proposed models for mechanism of the mitochondrial outer membrane permeabilization (MOMP). (A)** VDAC is the OMM component of the MPTP complex. In this model, pro-apoptotic proteins (Bax and Bad) interact with VDAC to accelerate its opening, whereas Bcl-X_L_ binds to VDAC directly to close it. VDAC can cause cyt c release indirectly through the swelling and rupture of the OMM. However, this model has problems of relatively small pore size and dispensability of VDAC. **(B)** In an anti-apoptotic state, anti-apoptotic molecules (HK II and Bcl-X_L_) bind to VDAC and keep it in open configuration with low-conductance for the exchange of adenine nucleotides, which maintains OMM integrity. HK II competes with Bcl-X_L_ for binding site of VDAC. In a pro-apoptotic state, HK II detachment from VDAC promotes binding of Bcl-X_L_ to VDAC, releasing Bax from Bcl-X_L_. Free Bax interacts with Bax/Bak to form pore structures for the release of cyt c. Bad interacts with Bcl-X_L_ on Bcl-X_L_/Bax and Bcl-X_L_/VDAC, which release Bax from Bcl-X_L_/Bax heterodimer to form Bax/Bak and displace Bcl-X_L_ from VDAC to be sensitized to Ca^2+^, respectively. In this model, tBid was shown to induce VDAC closure, reducing adenine nucleotide exchange and creating mitochondrial dysfunction, which may cause the MOMP.

Because VDAC is the most abundant protein in the OMM, VDAC has long been believed to be a potential OMM component of the MPTP (Figure 1A) (McCommis and Baines, 2012). As a part of the MPTP, VDAC was thought to cause the release of cytochrome c indirectly through the swelling and rupture of OMM. The electrical conductance properties of VDAC were similar to those of the MPTP (Szabo et al., 1993), and the MPTP and VDAC shared the stimuli, such as ROS, Ca^2+^, voltage, adenine nucleotide, and pH (Halestrap et al., 2004; Di Lisa et al., 2007). Moreover, previous experiments using anti-VDAC monoclonal antibodies provided additional supporting evidence that the MPT in isolated mitochondria was prevented by blocking VDAC's channel activity (Zheng et al., 2004).

Nevertheless, the concept of this model has been discredited because of several conflicting evidence. Firstly, the estimated pore size of VDAC, 2.2 ± 0.05 nm (Pavlov et al., 2001), does not seem to be large enough for the passage of cytochrome c, of which the diameter is up to 3 nm (Kinnally and Antonsson, 2007). Secondly, the closure of VDAC may not correspond to the closure of MPTP. It was shown that treatment of isolated mitochondria with G3139, an 18-mer phosphorothioate blocker of VDAC, resulted in VDAC closure, which caused an accumulation of intramitochondrial ROS and accelerated onset of the MPT (Tikunov et al., 2010). Rostovtseva et al. reported that the VDAC channel, even in the closed state, was still large enough to pass solutes up to 1.5 kDa in size, which indicated that the closed state of VDAC might be the same as the open state of the MPTP (Rostovtseva et al., 2005). They also concluded that VDAC did not form channels that mediate the flux of proteins through membranes. Lastly, recent experiment with VDAC-deficient null mitochondria showed the maintenance of MPT responses, suggesting that VDAC might be dispensable for MPT and at least not an essential component of the MPTP (Baines et al., 2007).

### VDAC as a separate pore for adenine nucleotide exchange

Several competing models have been proposed to overcome the weaknesses of aforementioned model (Shimizu et al., 2000; Zalk et al., 2005; Shoshan-Barmatz and Ben-Hail, 2012). At this time, the most viable model for the MPTP might be VDAC as a separate channel for exchange of mitochondrial metabolites, such as adenine nucleotide (Figure 1B) (McCommis and Baines, 2012). The concept of this new model fits quite well into the MPTP paradigm, even though lots of mechanisms are to be determined.

The exchange of metabolites across the OMM is crucial for coupled cellular respiration (Vander Heiden et al., 2001). In a pro-apoptotic state, pro-apoptotic modulators such as tBid were found to close VDAC, which inhibited metabolite exchange between mitochondria and cytosol. The resultant build-up of the metabolites in the intermembrane space may cause mitochondrial dysfunction, leading to the MOMP and the release of cytochrome c (Rostovtseva et al., 2004). However, the mechanisms of how mitochondrial dysfunction can induce the MOMP and specific OMM route for cytochrome c are still unknown (McCommis and Baines, 2012). On the contrary, in an anti-apoptotic state, anti-apoptotic agents such as HK and Bcl-X_L_ have been shown to bind to VDAC and keep it open configuration, sustaining the exchange of the metabolic anions including ATP/ADP (Vander Heiden et al., 2001; Mathupala et al., 2006). It has been proposed that the maintenance of a low conductance state of VDAC may prevent the MOMP (Fulda et al., 2010).

## Regulation of the MPTP

### HK II-VDAC interaction

HK catalyzes the first step of the glycolytic pathway where glucose is phosphorylated to glucose-6-phosphate (Mathupala et al., 2006). Among four isoforms of HK, HK II is known to be overexpressed in most neoplastic cells where it contributes to the proliferation and survival of tumor cells through enhancing aerobic glycolysis, i.e., Warburg effect. In addition to providing the precursor for glycolysis and biosynthesis of key metabolites, mitochondrial HK II is believed to play an important role in maintaining the OMM integrity through the interaction with VDAC and inhibiting mitochondrial-mediated apoptosis.

Binding of HK II to VDAC may inhibit the MPTP formation in at least two ways (Mathupala et al., 2006). Firstly, HK II binding could change the conformation of VDAC, which might in turn alter the conformation of ANT that is not conducive for formation of a MPTP. One of the possible roles of VDAC might be to keep the ANT in the c-conformation, in which the conversion of the ANT into the MPTP by Ca^2+^ is facilitated (Vyssokikh and Brdiczka, 2003). The preference of VDAC-bound HK II for mitochondria-derived ATP suggests that HK II binding to VDAC may induce a structural modification of VDAC for the passage of ATP (Vyssokikh and Brdiczka, 2003). Secondly, VDAC occupied by HK II is shown to prevent Bax and other pro-apoptotic proteins binding to it, which in turn prevents their oligomerization necessary for activation of the MPTP (Mathupala et al., 2006).

The importance of HK II-VDAC interaction as a key component of the regulation of the cellular apoptotic signaling cascades is further supported by the research of Shulga et al. (2009). They used the fifteen amino acid N-terminal peptide of HK II (N-HK II) which competes with the intact protein for binding to VDAC. Treatment of HCT-116 colon cancer cells with N-HK II peptide was found to re-localize HK II from the mitochondria to the cytosol. Furthermore, treatment of HCT-116 cells with cisplatin in combination with the N-HK II peptide was shown to cause a synergistic enhancement of cytotoxicity. These results suggest that detachment of HK II from the mitochondria might sensitize the mitochondria to the cisplatin-induced cell damage, leading to much enhanced cytotoxicity (Shulga et al., 2009).

Akt, also known as protein kinase B and the potent effector of anti-apoptotic stimuli in tumors, is another potent regulator of HK II-VDAC binding (Mathupala et al., 2006). Akt can directly phosphorylate HK II to enhance binding of HK II to the mitochondria (Elstrom et al., 2004).

### Bcl-2 family proteins

Another critical regulation of the cellular apoptosis via the MPTP is the balance between pro- and anti-apoptotic Bcl-2 family proteins (Gogvadze et al., 2009b). Bcl-2 family proteins have classically been comprised of three groups: anti-apoptotic multidomain proteins (Bcl-2, Bcl-X_L_, Bcl-W, Mcl-1, and A1A); pro-apoptotic multidomain proteins (Bax and Bak); pro-apoptotic BH3-only proteins (Bim, Bid, Bad, Bik, Hrk, Puma, and Noxa) (Kroemer et al., 2007). It is generally accepted that BH3-only proteins can induce apoptosis primarily by inhibiting anti-apoptotic Bcl-2 family proteins and derepressing Bax and Bak. For example, Bad may interact with Bcl-X_L_ and release Bax from Bcl-X_L_/Bax heterodimers (Willis et al., 2005). In addition, Roy et al. suggested a Bax/Bak-independent mechanism of Bad to sensitize the opening of the MPTP, which indicated that dephosphorylated Bad could interact with Bcl-X_L_ and displacement it from VDAC that led to the sensitization of the MPTP to Ca^2+^ (Roy et al., 2009). Whereas Bax is mostly cytosolic, Bak is known to reside on the OMM under normal circumstances. Although how Bax and Bak induce the MOMP remains elusive, it seems to be clear that Bax coalescence through translocation to the OMM and Bak activation promote mitochondrial fragmentation and cytochrome c release (Youle and Strasser, 2008).

Regarding HK II-VDAC interaction, HK II may compete with Bcl-X_L_ for VDAC binding site. Thus, HK II binding to VDAC releases Bcl-X_L_ from VDAC and facilitates binding of Bcl-X_L_ to Bax, which prevents Bax-Bax oligomerization or Bax-Bak interaction. However, HK II detachment from VDAC, for example, through phosphorylation of VDAC by glycogen synthase kinase 3β (GSK 3β), promotes binding of Bcl-X_L_ to VDAC, leaving Bax free from Bcl-X_L_. Free Bax interacts with Bak and/or Bax to form pore structures for the release of cytochrome c (Pastorino and Hoek, 2008). Interestingly, though all VDAC in this review indicate VDAC 1, Cheng et al. reported the distinct role of VDAC 2, a VDAC isoform present in low abundance that interacts specifically with the inactive conformer of Bak (Cheng et al., 2003). They showed cells deficient in VDAC 2, but not cells lacking VDAC 1, exhibited increased Bak oligomerization and were more susceptible to apoptotic death. In addition, they also indicated that BH3-only proteins displaced VDAC 2 from Bak, enabling Bak-Bak interaction and apoptosis.

### CyP-D as a regulator of HK II biding to VDAC

Several studies have shown that CyP-D is up-regulated in many human tumors and can function as an apoptosis repressor (Lin and Lechleiter, 2002; Schubert and Grimm, 2004). Growing number of evidence demonstrated that the anti-apoptotic regulation of CyP-D might be associated with the stabilization of HK II binding to mitochondria (Machida et al., 2006; Chiara et al., 2008). Inactivation of CyP-D with cyclosporine A or knock-down of the expression using siRNA was shown to release HK II from mitochondria. Because CyP-D is a mitochondrial matrix protein, an intermediate in the IMM between the OMM and matrix is necessary for its modulation of HK II binding to VDAC. ANT in the IMM could play this intermediation role (Chevrollier et al., 2005). However, Chiara et al. showed the opposing pro-apoptotic role of CyP-D in apoptosis (Chiara et al., 2008). They demonstrated that HK II detachment-triggering apoptosis might be associated with a disruption of the interaction of CyP-D with ANT. Furthermore, inhibition of CyP-D was shown to prevent the onset of the MPTP. Further investigation is needed to fully understand the role of CyP-D in apoptosis.

### ERK/GSK3/CyP-D transduction pathway

Recently, ERK/GSK3/CyP-D was proposed to be a transduction axis which was responsible for the regulation of the MPTP opening in tumor cells (Rasola et al., 2010; Traba et al., 2012). ERK was reported to be constitutively activated in mitochondria of several cancer cell types (Rasola et al., 2010). Recent work indicated that ERK activation prevented the release of apoptogenic proteins whereas the inhibition of ERK activity induced profound depletion in cellular ATP coincident with a loss of mitochondrial membrane potential (Monick et al., 2008). In addition, ERK activation was shown to inhibit GSK3-dependent phosphorylation of the MPTP regulator CyP-D and render cancer cells more refractory to the MPTP opening (Rasola et al., 2010).

## Targeting candidates

Again, cytochrome c release is the commitment step of mitochondria-mediated apoptosis. Therefore, the manipulation of mitochondrial channels responsible for the release of cytochrome c from intermembrane space could provide a window of opportunity for efficient and selective anti-cancer therapy. A variety of compounds that act via mitochondria, so called, mitocans, were shown to destabilize mitochondria and cause apoptosis in selected cancer cells (Ralph and Neuzil, 2009). Many of MPTP-targeting mitocans are currently under various stages of clinical development (Table 1).

**Table 1:** **Summary of the developmental status of anti-cancer agents targeting mitochondrial apoptotic machinery**.

**Agent**	**Target or mode of action**	**Phase of clinical development**	**References**
**MODULATORS OF THE Bcl-2 FAMILY PROTEINS**			
ABT-263	Bcl-2, Bcl-X_L_, Bcl-W	Phase II	Rudin et al., 2012
ABT-737	Bcl-2, Bcl-X_L_, Bcl-W	Pre-clinical (*in vitro* and *in vivo*)	Mason et al., 2008
AT-101 (gossypol)	Bcl-2, Bcl-X_L_, Bcl-W, Mcl-1	Phase II	Van Poznak et al., 2001
GX15-070 (obatoclax)	Bcl-2, Bcl-X_L_, Bcl-W, Mcl-1	Phase II	Parikh et al., 2010
HA14-1	Bcl-2	Pre-clinical (*in vitro* and in vivo)	Simonin et al., 2009
G3139 (oblimersen)	Bcl-2 mRNA antisense	Phase III	O'Brien et al., 2007
**METABOLIC INHIBITORS**			
Methyl jasmonate	HK II-VDAC interaction	Pre-clinical (*in vitro* and *in vivo*)	Goldin et al., 2008
3-Bromopyruvate	HK II-VDAC interaction	Pre-clinical (*in vitro* and *in vivo*)	Chen et al., 2009
HK II peptide	HK II-VDAC interaction	Pre-clinical (*in vitro*)	Chiara et al., 2008
**VDAC AND/OR ANT-TARGETING AGENTS**			
Arsenite trioxide (As_2_O_3_)	ANT ligand, ROS production	Phase IV	Powell et al., 2010
Lonidamine	ANT ligand	Phase IV	Di Cosimo et al., 2003; Oudard et al., 2003
Clodronate	ANT inhibitor	Phase III	Lehenkari et al., 2002
GSAO	ANT cross linker	Pre-clinical (*in vitro* and *in vivo*)	Don et al., 2003
FNQs	VDAC1	Pre-clinical (*in vitro* and *in vivo*)	Simamura et al., 2006
Erastin	VDAC2 and VDAC3	Phase I	NCT00528047
**ROS REGULATORS**			
Motexafin gadolinium	ROS production, Akt	Phase III	Mehta et al., 2009
Bismaleimido-hexane	ANT thiol oxidation	Pre-clinical (*in vitro*)	Palmeira and Wallace, 1997
Dithiodipyridine	ANT thiol oxidation	Pre-clinical (*in vitro* and *in vivo*)	Lifson et al., 2004
**RETINOIDS**			
All-trans-retinoid acid	ANT ligand	Pre-clinical (*in vitro* and *in vivo*)	Notario et al., 2003
CD437	MPTP	Pre-clinical (*in vitro*)	Marchetti et al., 1999; Belzacq et al., 2001
**NATURAL COMPOUNDS**			
Resveratrol	F1-ATPase	Phase II	Gledhill et al., 2007
Curcumin	Bax, Bcl-2, Bcl-X_L_, NF-κ B	Phase III	Carroll et al., 2011
Betulinic acid	MPTP	Pre-clinical (*in vitro*)	Fulda et al., 1997
Berberine	ANT ligand	Pre-clinical (*in vitro* and *in vivo*)	Pereira et al., 2008
α-tocopheryl succinate	Bax, ubiquinone-binding sites in respiratory complex II	Pre-clinical (*in vitro* and *in vivo*)	Dong et al., 2008
Honokiol	Cyclophilin D	Pre-clinical (*in vitro* and *in vivo*)	Arora et al., 2012

ANT, adenine nucleotide translocase; GSAO, 4-(N-(S-glutathionylacetyl)amino) phenylarsenoxide; CD437, 6-[3-(1-adamantyl)-4-hydroxyphenyl]-2-naphthalene carboxylic acid; HA14-1, 2-amino-6-bromo-4-(1-cyano-2-ethoxy-2-oxoethyl)-4H-chromene-3-carboxylate; HK, hexokinase; Mcl-1, myeloid cell leukemia sequence 1; MPTP, mitochondrial permeability transition pore; ROS, reactive oxygen species; VDAC, voltage-dependent anion channel. Modified and adapted by permission from Fulda et al. (2010) and Barbosa et al. (2012).

### Modulators of the Bcl-2 family proteins

#### ABT-737, ABT-263

The susceptibility of cancer cells to undergo apoptosis via MPTP is often determined by the balance between pro-apoptotic and anti-apoptotic Bcl-2 family proteins. BH3 mimetics, small molecules that have close structural or functional similarity to BH3-only proteins, may shift the balance toward pro-apoptotic members, initiating MOMP-dependent apoptosis (Fulda et al., 2010).

One of best characterized BH3 mimetics, ABT-737, binds to Bcl-2, Bcl-X_L_, and Bcl-W, releasing sequestered pro-apoptotic proteins and inducing cell death through the intrinsic apoptotic pathway. For example, ABT-737 can bind to interfere with the binding of Bcl-2 with pro-apoptotic proteins, releasing free form of pro-apoptotic proteins. Increased expression of Bcl-2 in tumor cells indicates that these cells might be “primed to die” because of the increased amount of sequestered pro-apoptotic proteins (Dejean et al., 2010). However, many cancers with overexpression of Bcl-2 exhibit a resistance to mitochondrial apoptotic cell death (Certo et al., 2006). This means that ABT-737 may act as an apoptosis sensitizer synergistically with chemotherapeutic agents to reverse the chemoresistance and selectively kill these cancer cells without serious toxicity (Hann et al., 2008; Kutuk and Letai, 2008). Nonetheless, Van Delft et al. showed the refractoriness of many types of cancer cells to ABT-737 possibly due to ABT-737's inability to target another pro-survival protein, Mcl-1(Van Delft et al., 2006). The expected advantage of targeting of both the Bcl-2 and Mcl-1 mechanisms of apoptosis resistance was demonstrated in various types of cancers (Bray et al., 2009; Zhang et al., 2012). To improve the clinical compliance of ABT-737, an orally available derivative, ABT-263, has been generated and now under investigation in various clinical trials commonly in combination with conventional chemotherapeutic agents or other molecular targeted agents in both solid and blood malignancies (Wilson et al., 2010; Rudin et al., 2012).

#### AT-101 (gossypol)

Gossypol, a natural phenolic compound in cotton plants, is shown to inhibit Bcl-2, Bcl-X_L_, Bcl-W, Mcl-1 (Azmi and Mohammad, 2009). Gossypol showed clinical activity in a phase I trial against prostate cancer and currently under investigation (Liu et al., 2009). Although gossypol exhibited a possible survival benefit in randomized phase II study for second-line treatment of non-small cell lung cancer in combination with docetaxel (Ready et al., 2011), Baggstrom et al. failed to show the efficacy of gossypol in patients with recurrent chemosensitive small cell lung cancer (Baggstrom et al., 2011).

#### Gx15-070 (obatoclax)

Obatoclax, a small molecule indole bipyrrole compound, also inhibits Bcl-2, Bcl-X_L_, Bcl-W, Mcl-1 (O'Brien et al., 2009). Obatoclax was demonstrated to efficiently disrupt the interaction between Bak and Mcl-1, overcoming the mcl-1 dependent resistance to the proteasome inhibitor bortezomib against multiple myeloma (Nguyen et al., 2007).

#### HA14-1

HA14-1, an organic compound, was shown to specifically inhibit Bcl-2 (Wang et al., 2000). Although HA14-1 was reported to increase the sensitivity of cancer cells to chemotherapy or radiotherapy, the clinical trials with HA14-1 have not yet been initiated (Manero et al., 2006).

#### G3139 (oblimersen)

Oblimersen, an 18-mer phosphorothioate Bcl-2 mRNA anti-sense, anneals to the initiation codon region of Bcl-2 mRNA, thereby inhibiting Bcl-2 biosynthesis (Fulda et al., 2010). Oblimersen was also shown to directly bind and reduce the channel conductance of VDAC (Tan et al., 2007).

### Disruptors of the HK II-VDAC interaction

HK II-VDAC interactions are now believed to be crucial also for promoting cancer survival via modulation of signaling events related to apoptosis. Considering that HK II is frequently overexpressed in cancers and HK-VDAC binding is tighter in cancer cells than that in normal counterparts, disruption of the HK II-VDAC interaction could offer another basis for a novel selective anti-cancer strategy (Pastorino et al., 2005).

#### Methyl jasmonate

Methyl jasmonate, a plant-derived small molecule, is shown to exhibit anti-cancer activity via specific binding to HK II and disrupting the HK II-VDAC interaction, thereby favoring MOMP in cancer cell lines (Goldin et al., 2008). Goldin et al. also reported that methyl jasmonate could have cancer-selective effects because many types of cancer cells overexpressed HK II with excessive binding to mitochondria compared with their normal counterpart. Actually there are two additional anti-cancer mechanisms of methyl jasmonate: induction of severe ATP depletion in cancer cells via mitochondrial perturbation and induction of apoptosis in lung carcinoma cells via the generation of hydrogen peroxide and pro-apoptotic Bcl-2 family proteins (Cohen and Flescher, 2009).

#### 3-bromopyruvate

The pyruvate analog, 3-bromopyruvate, is an alkylating agent and a potent inhibitor of glycolysis (Ganapathy-Kanniappan et al., 2010). 3-Bromopyruvate is known to inhibit energy metabolism of glycolysis-dependent tumor cells and trigger cell death presumably through depletion of cellular ATP (Kim et al., 2007). Moreover, 3-bromopyruvate was recently shown to cause a covalent modification of HK II protein and directly trigger its dissociation from mitochondria, leading to specific release of apoptosis inducing factor (AIF) from the mitochondria to cytosol and eventual cell death (Chen et al., 2009).

#### Clotrimazole

Clotrimazole was shown to induce HK II detachment from mitochondria of many cancer cells and prompt a concentration-dependent cell death (Pastorino et al., 2002; Machida et al., 2006). However, the toxic effects of clotrimazole on mitochondria appeared to be through the inhibition of mitochondrial respiration by binding to a molecular target other than HK II (Chiara et al., 2008). Therefore, the non-specific toxic effects of clotrimazole are likely to cause significant side effects, which might discourage its clinical use as an anti-cancer drug.

#### HK II-based peptides

As mentioned above, newly designed oligopeptides corresponding to the N-terminal hydrophobic domain of HK II were shown to compete with the intact HK II for binding to VDAC and effectively displace HK II from VDAC (Pastorino et al., 2002). Unlike clotrimazole, the HK II-based peptide is expected to exhibit tumor cell specificity because it does not affect mitochondrial respiration (Chiara et al., 2008). Clinical trials for HK II-based peptide are warranted.

### ANT inhibitors

#### Arsenite trioxide (As_2_O_3_)

Arsenite is recently proved to be highly effective in treating acute promyelocytic leukemia (APL) (Powell et al., 2010). One of the key mechanisms proposed was an apoptotic effect via the opening of the MPTP (Larochette et al., 1999). Arsenite was shown to dissipate the mitochondrial membrane potential, while the pro-apoptotic effect of arsenite was inhibited by Bcl-2 overexpression (Larochette et al., 1999). It has been found that ANT is one of the targets of arsenite within MPTP (Belzacq et al., 2001). Cell free systems of apoptosis have revealed that arsenite induces the permeabilization of ANT proteoliposomes and requires mitochondria to trigger nuclear apoptosis *in vitro* (Belzacq et al., 2001).

#### Lonidamine

Lonidamine, an indazole carboxylate, has been demonstrated to inhibit ANT and trigger mitochondria-mediated apoptosis (Fulda et al., 2010). It was found that lonidamine induced the permeabilization of liposomes containing ANT, while had no effect on protein-free liposomes (Belzacq et al., 2001). Despite the cytostatic effect in recurrent glioblastoma multiforme and the additional improvement of response rate in epirubicin treatment for the patients with advanced breast cancer, lonidamine was shown to be toxic to non-tumor tissues, for example, causing hepatotoxicity (Barbosa et al., 2012). Nevertheless, lonidamine is currently undergoing phase III/IV clinical trials (Table 1).

#### Clodronate

Clodranate, a nitrogen-free bisphosphonate, can also act as competitive ANT inhibitor, leading to the dissipation of the mitochondrial membrane potential and apoptosis (Fulda et al., 2010). Although the underlying mechanisms are still elusive, it was shown that the addition of clodronate to postoperative adjuvant therapy in breast cancer patients improved the overall survival outcomes (Diel et al., 2008).

#### 4-(N-(S-glutathionylacetyl)amino) phenylarsenoxide (GSAO)

GSAO was shown to cross-link cysteine residues of ANT and inhibit the exchange of ATP/ADP through ANT, leading to mitochondrial membrane depolarization and apoptosis (Don et al., 2003). Interestingly, GSAO seems to be a promising anti-cancer drug since GSAO is shown to inhibit tumor angiogenesis by selectively targeting mitochondria in proliferating endothelial cells presumably due to the higher amounts of mitochondria in endothelial cells than in tumor cells (Don et al., 2003).

#### Furanonaphthoquinones (FNQs)

FNQs, isolated from the inner bark of tropical trees, were shown to target VDAC1 (Simamura et al., 2006). FNQs might induce apoptosis via the NADH-dependent production of ROS, leading to collapse of the mitochondrial membrane potential and caspase 9 activation (Simamura et al., 2006). The ROS production of FNQs was increased upon VDAC1 overexpression.

#### Erastin

Erastin was shown to bind VDAC2 and 3 to induce NADH-dependent oxidative cell death by inducing RAS-RAF-MEK pathway (Pathania et al., 2009). The erastin analogue, PRLX 93936, is currently undergoing phase I clinical trial (Table 1).

#### All-trans-retinoid acid

All-*trans*-retinoid acid and retinoid-related compounds, CD437 (6-[3-(1-adamantyl)-4-hydroxyphenyl]-2-naphthalene carboxylic acid) are well known to induce clinical remission in patients with APL (Slack and Rusiniak, 2000). In addition to the stimulation of the expression of retinoic acid receptor-responsive genes, these retinoids was recently shown to trigger ANT-dependent MPT (Belzacq et al., 2001; Notario et al., 2003).

### ROS regulators

#### Motexafin gadolinium

Motexafin gadolinium (MGd) is an expanded porphyrin which displays an elevated oxidizing potential, thereby triggering excess generation of ROS (Fulda et al., 2010). MGd is known as a radio- and chemo-sensitizer because of the preferential accumulation in cancer cells (Magda and Miller, 2006). Intriguingly, pro-apoptotic effect of MGd was shown to be enhanced by combining it with Akt phosphorylation inhibitors such as celecoxib or docetaxel (Ramos et al., 2006). It suggests that modulation of the levels of phosphorylated Akt may be one of the modes of action of MGd and the synergistic cytotoxicity can be induced by the combination of MGd with inhibitors of Akt phosphorylation (Ramos et al., 2006).

#### Thiol crosslinking agents

Bismaleimido-hexane (BMH) and dithiodipyridine (DTDP) can cause thiol oxidation of a critical cysteine residue (Cys 56) of ANT, through which BMH and DTDP may induce MOMP and cell death irrespective of the expression level of Bcl-2 (Costantini et al., 2000). Moreover, recombinant Bcl-2 was found to fail to prevent thiol modification of ANT, suggesting that both thiol crosslinkers could bypass Bcl-2-mediated cytoprotection (Costantini et al., 2000).

### Natural compounds

#### Resveratrol

Resveratrol, a polyphenolic compound from grapes and wine, can inhibit mitochondrial ATP synthesis and trigger MOMP (Fulda et al., 2010). Recently, one of the synthetic resveratrol analogues, HS-1793, was shown to induce collapse of mitochondrial membrane potential and cytochrome c release in murine breast cancer cell lines (Kim et al., 2012). In addition, HS-1793 was also demonstrated to circumvent Bcl-2-mediated apoptosis resistance in leukemia cells (Jeong et al., 2009). Resveratrol is currently undergoing phase I/II clinical trials (Table 1).

#### Curcumin

Curcumin is a major constituent of turmeric powder from the plant Curcuma longa. Among several anti-cancer mechanisms of curcumin are the modulation of Bcl-2 family proteins and cellular ROS, inhibition of the NF-κ B survival pathway, and inhibition of cyclooxygenase-2 (Divya and Pillai, 2006; Singh and Khar, 2006; Madden et al., 2009; Pinlaor et al., 2009; Shehzad et al., 2010). Curcumin is currently undergoing phase II/III clinical trials.

#### Betulinic acid

Betulinic acid, a natural pentacyclic triterpenoid of the lupane class, is known to trigger mitochondrial apoptosis in cancer cells (Fulda et al., 2010). Betulinic acid not only directly triggers MOMP but also modulates Bcl-2 family proteins (Fulda et al., 1997; Selzer et al., 2002).

#### Berberine

Berberine, an alkaloid derived from plants of the Berberidaceae family, has been shown to exert direct effects on mitochondria, including the interaction with ANT (Pereira et al., 2008). Several other mechanisms underlying the induction of apoptosis by berberine includes the alterations in the Bcl-2/Bax ratio, ROS production, a decrease in mitochondrial membrane potential, and cytochrome c release (Fulda et al., 2010).

#### α-tocopheryl succinate (α-TOS)

α-TOS, a vitamin E analogue, competes with ubiquinone in binding to Q sites of respiratory complex II, which results in the displacement of ubiquinone from complex II, disrupts the electron flux, and consequently generating ROS (Dong et al., 2008). In the cytosol, ROS catalyze the formation of disulfide bridges between Bax monomers, causing a conformational change for dimerization which is followed by Bax translocation to the OMM to form channels (Neuzil et al., 2006). In addition, α-TOS binds to Bcl-2 and Bcl-X_L_ on the OMM to prevent them trapping Bax, allowing free Bax to form OMM channels (Neuzil et al., 2006). The same group recently proposed another mechanism of α-TOS-induced apoptosis, which involved Noxa-Bak axis (Prochazka et al., 2010). The tumor selectivity of α-TOS due to its ester structure and preferential triggering apoptosis in proliferating endothelial cells form very promising *in vitro* data, which warrant further clinical trials with α-TOS (Neuzil et al., 2001; Dong et al., 2007).

#### Honokiol

Honokiol is a small molecule polyphenol isolated from the genus Magnolia (Fried and Arbiser, 2009). Recently, the antiangiogenic and antitumor activities of honokiol have been reported in preclinical models. Major mechanisms of action of honokiol include the induction of CyP-D, potentiating the MPTP. Importantly, honokiol-induced CyP-D-regulated cell death was shown to be able to overcome Bcl-2 and Bcl-XL-mediated apoptotic resistance in primary human leukemia cells (Li et al., 2007). These promising preclinical data may warrant the development of clinical trials of honokiol.

### Inhibition of ERK/GSK3/CyP-D transduction pathway

Again, GSK3 plays a role in the opening of the MPTP through the phosphorylation of CyP-D. In 206 osteosarcoma ρ° cells with an extreme Warburg phenotype, constitutively active ERK was shown to oppose this signaling by phosphorylating and inhibiting GSK3 (Masgras et al., 2012). Starvation through serum and glucose depletion resulted in ERK inhibition and GSK3 activation in ρ° cells. Through the activation of ERK/GSK3/CyP-D transduction pathway, starvation could induce 206 ρ° cells to undergo a rapid mitochondrial depolarization (Masgras et al., 2012). Therefore, starvation might be another way to induce MPTP opening in the glycolysis-dependent tumor cells.

## Conclusions

Mitochondria have pivotal opposing roles in energy generation for cell survival and cytochrome c release for apoptotic cell death. Although the concept of the MPTP is still evolving, mounting evidence indicates that the MPTP is directly responsible for cytochrome c release, leading to apoptotic cell death. Targeting the MPTP for cancer treatment has two advantages: tumor specificity and bypass of the resistance mechanisms. Metabolic reprogramming and mitochondrial alterations in cancer cells could provide important clues for developing tumor-specific anti-cancer agents. MOMP finally occurs as a consequence of upstream pro-apoptotic signaling events, which are frequently deregulated in many cancers and which become resistant to most classical anti-cancer agents that target upstream regulators of MOMP (Fulda et al., 2010). Because of this convergence of apoptotic signaling on the MOMP, anti-cancer drugs that directly target the MPTP could have the potential to bypass the resistance mechanisms of cancer. Therefore, better understanding at a molecular composition of the MPTP will provide clues for effective and selective therapeutic strategies for the treatment of cancer. Further studies are warranted to elucidate the mechanism of MOMP through the MPTP.

### Conflict of interest statement

The authors declare that the research was conducted in the absence of any commercial or financial relationships that could be construed as a potential conflict of interest.
